# Targeting the Galectin-1/Ras Interaction for Treating Malignant Peripheral Nerve Sheath Tumors

**DOI:** 10.21203/rs.3.rs-5263500/v1

**Published:** 2024-10-16

**Authors:** Hsiao-Chi Wang, Keila E. Torres, Roger Xia, Marcio Malogolowkin, Ssu-Wei Hsu, Ching-Hsien Chen, Tsung-Chieh Shih

**Affiliations:** 1 Department of Research and Development, Kibio Inc; Houston, Texas, USA.; 2 Department of Surgical Oncology, The University of Texas MD Anderson Cancer Center; Houston, Texas, USA.; 3 Department of Biomedical Data Science, Stanford University; Stanford, California, USA.; 4 Division of Pediatric Hematology-Oncology, UC Davis Children’s Hospital; Sacramento, California, USA.; 5 Divisions of Nephrology and Pulmonary, Critical Care and Sleep Medicine, Department of Internal Medicine, University of California at Davis; Davis, California, USA.; 6 Comprehensive Cancer Center, University of California at Davis; Davis, California, USA; 7 Department of Translational Molecular Pathology, The University of Texas MD Anderson Cancer Center; Houston, Texas, USA.

## Abstract

**Background:**

Neurofibromatosis type 1 (NF1) is a common inherited neurological disorder that can lead to the development of malignant peripheral nerve sheath tumors (MPNSTs), a highly aggressive form of sarcoma. Current treatment options for MPNSTs are limited, with poor prognosis and high recurrence rates. This study aims to explore the potential of targeting the Galectin-1 (Gal-1) and Ras interaction as a novel therapeutic strategy for MPNSTs.

**Methods:**

Molecular docking simulations were conducted to identify specific residues involved in the Gal-1 and H-Ras(G12V) interaction. LLS30, a compound designed to target the Ras binding pocket on Gal-1, was developed and tested. The efficacy of LLS30 was evaluated through in vitro assays, including cell viability, apoptosis, and co-immunoprecipitation studies, as well as in vivo assays using orthotopic MPNST xenograft and experimental lung metastasis models. Transcriptome sequencing was performed to analyze the impact of LLS30 on gene expression and signaling pathways.

**Results:**

Molecular docking revealed key residues involved in the Gal-1/Ras interaction, and LLS30 was shown to bind to these residues, disrupting the interaction. LLS30 treatment resulted in Ras delocalization from the plasma membrane and suppression of the Ras/Erk signaling pathway. *In vitro*, LLS30 significantly reduced MPNST cell proliferation and induced apoptosis. *In vivo*, LLS30 demonstrated potent anti-tumor activity, reducing tumor burden and metastasis while improving survival in animal models. Transcriptome analysis showed that LLS30 downregulates critical pathways, including KRAS signaling and epithelial-mesenchymal transition (EMT).

**Conclusions:**

Interference with the Gal-1/Ras interaction could lead to suppression of the Ras signaling pathway. LLS30 effectively disrupts the Gal-1/Ras interaction, resulting in significant anti-tumor and anti-metastatic effects in MPNST models. These findings indicated that targeting Gal-1 with LLS30 offers a promising therapeutic approach for treating MPNSTs and may also be applicable to other malignancies where Gal-1 and Ras are key oncogenic drivers.

## Background

Neurofibromatosis type 1 (NF1) is a tumor that grows along nerves in the skin, brain, and other parts of the body. NF1 is the most common inherited neurological disorder, affecting approximately 1 in 3,000 people throughout the world(1). The primary nerve-associated tumors in NF1 are neurofibromas, which are mostly benign and broadly classified into dermal and plexiform subtypes(2, 3). Although neurofibromas are benign tumors, plexiform neurofibromas can transform into malignant peripheral nerve sheath tumors (MPNSTs) known as malignant schwannomas or neurofibrosarcomas. Between 8% and 13% of people with NF1 will get an MPNST in their lifetime so that approximately 800,000 people worldwide are affected per year(4–6). Approximately 20% of cases are diagnosed in the pediatric population(7).

An MPNST is considered a high-grade sarcoma with the potential to recur and metastasize. Currently, surgical resection is the main treatment for this disorder(8). Unfortunately, complete surgical removal is almost impossible because tumors may develop in deep peripheral nerves or roots. Surgical resection can even cause disability. MPNSTs pose significant management challenges. For unresectable or metastatic diseases, chemotherapeutic drugs are only marginally effective (with a response rate of <21%), and initial responses to therapy are usually short-lived with a recurrence rate of 40–65%, followed by rapid progression and death(9). As such, 5-year overall survival rates remain low (the 5-year survival is <50%)(10, 11). However, there are no effective systemic therapies for MPNST patients.

NF1 is a common genetic disorder caused by defects in the NF1 gene, which encodes the protein neurofibromin. Neurofibromin functions partly as a Ras GTPase-activating protein (GAP), converting active Ras-GTP to inactive Ras-GDP(12). By accelerating the hydrolysis of GTP to GDP, neurofibromin acts as a negative regulator of Ras(13). Mutations in the NF1 gene in neurofibromas and MPNST result in elevated levels of active Ras-GTP(14, 15). Ras is initially translated on ribosomes as an inactive protein and attaches to the cell membrane after post-translational modification (prenylation and palmitoylation) and further stabilized by interacting with chaperon proteins such as PDEδ and RAP1GDS1(16, 17). Activated Ras at the plasma membrane is crucial for triggering oncogenic signaling pathways. Therefore, targeting Ras chaperones to disrupt Ras membrane association and subsequently inhibit its oncogenic function holds significant promise for the development and application of effective treatments for MPNST.

Galectin-1 (Gal-1), a 14 kDa lectin, is one of a galectin family with an affinity for β-galactosides. The 135 kDa Gal-1 protein is encoded by the gene *LGALS1* at 22q13.1. Increased Gal-1 expression by tumor and connective tissue is regarded as a sign of malignant progression and often correlates with aggressiveness and a metastatic phenotype(18–20). Gal-1-binding proteins that have been discovered include H-Ras, integrins, laminins, fibronectin, vitronectin, osteopontin, neuropilin-1, CD44, CD146, and CD326, among others(21, 22). Our previous study has shown elevated Gal-1 levels in MPNST patients and cells, with Gal-1 knockdown leading to Ras pathway suppression and inhibition of cancer cell proliferation both *in vitro* and *in vivo*(23). Mechanistically, Gal-1, functioning as a chaperone for Ras, interacts and stabilizes activated Ras at the plasma membrane thereby resulting in the activation of the Ras oncogenic signaling pathway(23). Disruption of Gal-1/H-Ras(G12V) interaction could be an effective strategy for treating MPNSTs. However, the specific residues involved in Gal-1/H-Ras(G12V) interaction are not well understood.

In this study, we have identified the exact residues in both H-Ras(G12V) and Gal-1 that mediate this interaction. In addition, we reveal for the first time that the Gal-1 inhibitor LLS30, which binds to the same pocket on Gal-1 where H-Ras(G12V) binds, is able to block the Gal-1/H-Ras(G12V) interaction, effectively disrupting this critical oncogenic pathway. These findings highlighted the importance of Gal-1 in MPNSTs and suggested that inhibiting Gal-1/H-Ras(G12V) interaction could be a promising therapeutic strategy for this aggressive cancer.

## Methods

### Cell lines

The cell lines used in this study include MPNST-derived cell lines NF02.2 and NF96.2 and HEK293T. These cells were cultured in DMEM supplemented with 10% fetal bovine serum and 1% penicillin/streptomycin and grown in 5% CO_2_ at 37°C. Routine monthly testing for Mycoplasma contamination was conducted.

### Cell viability and apoptosis assay

To assess cell viability, 5 × 10^3^ NF96.2 and NF2.2 cells were seeded per well into 96-well plates and allowed to attach for 24 hours before drug treatment for 72 hours. Following the 24-hour incubation period, the medium was removed, and the cells were treated with specified concentrations of LLS30 or OTX008. LLS30 and OTX008 stock solutions (10 mM) were initially prepared in 100% DMSO (100X concentration). A working solution of 100 μM LLS30 in 1% DMSO was obtained by diluting the stock solution 1:100 with cell culture medium, which was then subjected to two-fold serial dilutions in cell culture medium. Cell viability was determined at indicated time points using the MTT assay. For apoptosis assessment, caspase-3/7 activity was measured following treatment with 0.05% DMSO, 5 μM LLS30 for 12 and 24 hours using a luminescent caspase-Glo 3/7 assay kit (Promega).

### Immunoblotting analysis

The immunoblot assay was conducted following the protocol described earlier(23). Briefly, cells were lysed in RIPA buffer and incubated on ice for 20 minutes. Total cell lysates were collected after centrifugation and quantified by BCA assay. Lysates (20 μg each) were boiled in 2X Laemmli SDS-PAGE sample buffer, separated on 12% SDS-PAGE gels, and transferred to PVDF membrane. After blocking with 10% non-fat dried milk in Tris-buffered saline, membranes were incubated overnight at 4°C with specific primary antibodies against phospho Erk (Thr202/Tyr204, Cell Signaling), Erk (Cell Signaling) or beta-actin (Cell Signaling). Following three washes with TBS-T, membranes were incubated with HRP-linked secondary antibodies at 37°C for 1 hour. Chemiluminescence signal was detected using ECL substrate and a CCD camera.

### Protein-protein docking simulation

The 3D structures of the proteins H-Ras(G12V) and Gal-1 were downloaded from the RCSB PDB Database (https://www1.rcsb.org/), with the PDB ID for H-Ras (G12V) being 4EFM and for Gal-1 being 6F83. Protein-protein docking in ClusPro1 was utilized for molecular docking simulation and predicting the binding affinity of H-Ras(G12V) with Gal-1. Gal-1 was set as the ligand and H-Ras(G12V) as the receptor for protein docking. The ligand underwent 70,000 rotations, with translations along the x, y, and z axes relative to the receptor on a grid during each rotation. The translation yielding the best score from each rotation was selected. Out of 70,000 rotations, 1000 rotation/translation combinations with the lowest scores were chosen. Subsequently, a greedy clustering was conducted on these 1000 ligand positions using a 9 Å C-alpha RMSD radius to identify positions with the greatest number of neighboring ligands. The top ten cluster centers with the most cluster members were then retrieved and visually inspected one by one. Further evaluation was conducted on the intermolecular contacts from the most probable poses. The docked structures and interface residues were analyzed using MOE2, and molecular graphics were generated using PyMOL.

### Plasmid construction and protein production and purification

The pcDNA3.1/His-Gal-1 plasmid was commercially purchased from Gene Script, with sequences encoding Gal-1 proteins based on GenBank (NM_002305). Inverse PCR Mutagenesis was used to introduce mutations D123A in previously cloned sequences with the Phusion Site-Directed Mutagenesis Kit (ThermoScientific). The presence of the correct mutation was confirmed via sequencing. HEK293T cells were transiently transfected with the plasmids pcDNA3.1/His-Gal-1 and pcDNA3.1/His-Gal-1 D123A using Lipofectamine 2000 transfection reagent (Life Technologies) according to the manufacturer’s protocol. After 5 days post transfection, the cell pellet was collected by centrifugation at 400× g for 5 minutes, and subsequently, Gal-1 wild type (WT) and Gal-1 D123A mutant (Mut) proteins were purified using ProBond^™^ Nickel-Chelating Resin, as per the manufacturer’s instructions. The purity of the obtained fraction was assessed by SDS-PAGE analysis, with quantification performed using the Bradford method.

### Pull down assay

50 μL streptavidin agarose gel slurry (Pierce) was mixed with 50 μg biotinylated LLS30 in a spin column and incubated for 4 hours at 4°C. Subsequently, the sepharose slurry was added to 20 μg of whole cell lysate (WCL) from NF96.2 cells or 2 μg of Gal-1 WT or Gal-1 Mut, and incubated with rotary agitation overnight at 4°C. The sepharose was washed with PBS, and then bound proteins were eluted with 150 mM glycine, pH 2.5, for 10 minutes. The eluent was then neutralized by adding 10 μL neutralization buffer (Tris, pH 8.0) and subjected to immunoblotting to assess the Gal-1.

### Co-immunoprecipitation assay

2 × 10^6^ NF96.2 cells were first treated overnight with either LLS30 at 2 μM or DMSO at 0.02%, followed by membrane protein extraction using the ProteoExtract^®^ Native Membrane Protein Extraction Kit (Millipore). For immunoprecipitation, 10 μL of anti-Gal-1 antibody (Abcam) was gently mixed with 100 μL of Protein A/G Sepharose slurry (Abcam) and incubated for 4 hours at 4°C. The Sepharose slurry was then added to the protein mixtures and subjected to rotary agitation overnight at 4°C. After washing the sepharose with PBS, bound proteins were eluted with 150 mM glycine (pH 2.5) for 10 minutes. The eluent was neutralized by adding 10 mL of neutralization buffer (Tris, pH 8.0) and subjected to immunoblotting to assess Ras.

### In vivo animal assays

Animal experiments in this study were approved by the campus Institutional Animal Care and Use Committee (IACUC). LLS30 stock solutions (6X) were prepared in 50% absolute alcohol and 50% Tween 80 to make 15 mg/ml. Prior to administration, each was diluted with saline to produce 2.5 mg/mL solutions. Male congenital athymic BALB/c nude (nu/nu) mice were purchased from the Jackson laboratory. For MPNST orthotopic xenograft mouse models, mice were anesthetized with isoflurane, and the sciatic nerves were exposed bilaterally at mid-thigh. A cell suspension (5 × 10^5^ cells in 5 μL) was gradually injected into the sciatic nerve using a Microliter Syringe Model 701 N. The surgical site was closed with tissue adhesive (surgical glue), and the recovered mouse was returned to specific pathogen-free housing. Orthotopic tumors became palpable 6 weeks after injection. Mice were randomly divided into two groups (N=6) and intraperitoneally given (1) vehicle (8.7% alcohol/8.7% Tween 80), (2) 10 mg/kg LLS30 daily via intravenous administration for 14 consecutive days. Two weeks after treatment, bioluminescence signals were detected by the IVIS 200 Imaging System (Caliper LifeSciences), five minutes after intraperitoneal injection of 100 mg/kg D-luciferin. Quantification of tumor signals in mice with Aura software. After quantification, mice were then sacrificed, and tumors were excised for the activated Ras assay. For the lung metastasis model, 1 × 10^6^ luciferase-tagged NF96.2 cells were intravenously injected into nude mice, followed by LLS30 treatment (5 mg/kg once daily for 5 days) two weeks later. Eight weeks after implantation, bioluminescence signals were detected by the IVIS 200 Imaging System (Caliper LifeSciences), five minutes following intraperitoneal injection of 100 mg/kg D-luciferin.

### Ras activation assay

Activated Ras was detected using the Ras activation assay kit (Millipore) according to the manufacturer’s instructions. Briefly, xenografts were excised and lysed with a tissue homogenizer, and Raf-1 Ras-binding domain (RBD)-agarose beads were added to 200 μg of tissue lysates for 30 minutes at 4°C. Following centrifugation at 14,000×g for 10 seconds at 4°C and subsequent washing, the agarose-bound Ras was incubated in 2X Laemmli reducing sample buffer (126 mM Tris/HCl, 20% glycerol, 4% SDS, 0.02% bromophenol blue). The samples were then resolved by SDS-PAGE and detected by immunoblotting using an anti-Ras antibody (Millipore).

### Transcriptome sequencing and enrichment analysis

The RNA-seq data analysis involved extracting total RNA from both control and LLS30-treated NF96.2 cells using the PureLink RNA Mini Kit (Invitrogen) following the manufacturer’s instructions. RNA quality was evaluated using the Agilent 2100 Bioanalyzer system (Agilent Technologies). The mRNA sequencing library was prepared and paired-end sequencing was conducted using the Illumina HiSeq 4000 Sequencing System. Differentially expressed genes (DEGs) were identified with a 1.5-fold change and with a significance level of *p* < 0.05. Volcano plots were generated using the Sigmaplot software. Hallmark gene set analyses were performed on the Enrichr website (https://maayanlab.cloud/Enrichr/) using a list of gene symbols.

### Gene set enrichment analysis (GSEA)

GSEA was conducted using the Java desktop software (http://software.broadinstitute.org/gsea/index.jsp), following previously described methods(24). Genes were ranked based on shrunken limma log_2_ fold changes, and the GSEA tool was utilized in ‘pre-ranked’ mode with all default settings.

### Statistics analysis

*In vitro* experiments were conducted in triplicate across two independent experiments, and the results are presented as the mean ± SD. Survival curves for the control and LLS30 treatment groups were estimated separately using the Kaplan-Meier method and statistically compared with the log-rank test. The student’s t-test (two-tailed) was employed for comparing datasets between two groups with similar variance. *p* value < 0.05 was considered indicative of a statistically significant difference. Statistical differences, when compared with controls, are denoted as * (*p* < 0.05), ** (*p* < 0.01), or *** (*p* < 0.001).

### Data Availability

The data generated in this study are available upon request from the corresponding author.

## Results

### Molecular dynamics simulation of Gal-1, Ras, and LLS30 interactions

**Fig. 1.**
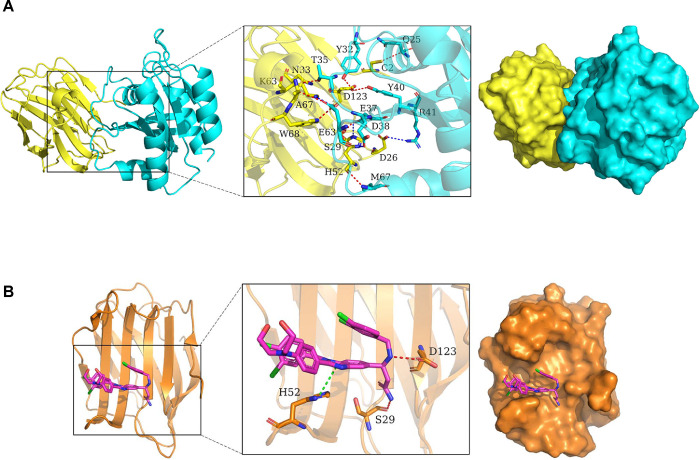
Molecular dynamics simulation of Gal-1, Ras, and LLS30 interactions. (A) The 3D binding model of Gal-1 with H-RAS(G12V). Gal-1 is colored with yellow, H-Ras(G12V) with cyan. The residues in Gal-1 are shown as yellow sticks, the residues in H-Ras(G12V) are shown as greencyan sticks. Red dashes indicate hydrogen bond interactions, while blue dashes represent salt bridges. (B) The 3D binding model of Gal-1 with LLS30. Gal-1 is colored with orange, LLS30 with magentas. The residues in Gal-1 are depicted as orange sticks. Red dashes represent hydrogen bond interaction while green dashes represent Pi-Pi conjugate.

Both our studies and others have indicated that Gal-1 functions as a chaperone for Ras and is necessary for Ras membrane localization, the primary site for activation of Ras(23, 25). However, the specific residues for this interaction remain unclear. To investigate the binding mode of H-Ras(G12V) with Gal-1, docking simulation studies were carried out. The interaction between H-Ras(G12V) with Gal-1 is shown in Fig. 1A. The contact list between H-Ras(G12V) with Gal-1 is shown in Table 1. Docking simulation studies indicates that the residues Gln25, Tyr32, Thr35, Asp38, Tyr40, Glu63 and Met67 in H-Ras(G12V) are involved in binding with Cys2, Asp123, Asn33, Ser29, Ala67, Trp68 and His52 in Gal-1 through hydrogen bond interactions. The residues Glu37, Asp38, Arg41 and Glu63 in H-Ras(G12V) are involved in binding with His52, Asp26, Lys63 through salt bridges. Given the critical role of Gal-1 in Ras activation, our objective was to disrupt the Gal-1/Ras interaction as a potential therapeutic strategy. Targeting the Ras binding pocket on Gal-1 may effectively block the interaction between Gal-1 and Ras. To explore this possibility, we tested LLS30 in this study to assess its ability to bind to the same pocket on Gal-1 where H-Ras(G12V) interacts. Molecular docking studies revealed that the aromatic groups of LLS30 are positioned within the hydrophobic core of Gal-1’s binding pocket, where residues His52, Ser29, and Asp123 play crucial roles in interacting with LLS30 through Pi-Pi stacking and hydrogen bonding (Fig. 1B). These findings from the molecular docking studies indicate that LLS30 has the potential to interfere with the Gal-1/Ras interaction by binding to key residues within Gal-1’s binding pocket.

### LLS30 disrupts Gal-1–Ras interactions and resulting delocalization of RAS

**Fig. 2.**
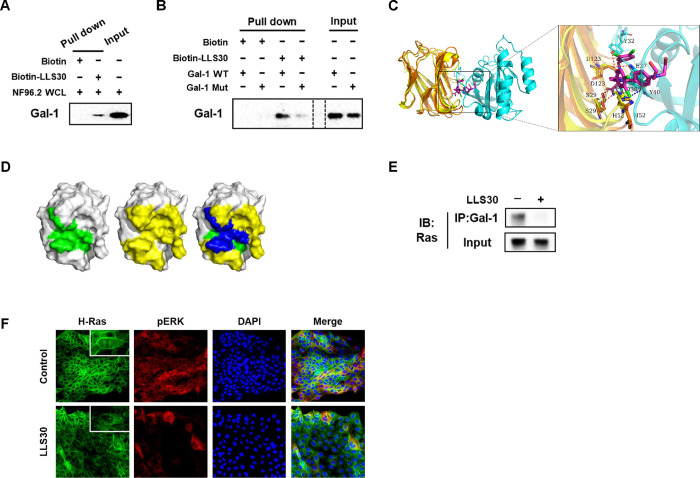
LLS30 interacts with Gal-1 and disrupts Gal-1/Ras interactions in MPNST Cells. (A) The pull-down assay confirmed the direct interaction between LLS30 and Gal-1 from NF96.2 whole cell lysate (WCL). Biotin-LLS30 conjugated with streptavidin-agarose beads was incubated with NF96.2 WCL to pull down interacting proteins. After thorough washing to remove non-specifically bound proteins, the proteins bound to the beads were eluted and confirmed by immunoblots using an antibody against Gal-1. The input lane represents NF96.2 WCL without pull-down, serving as an input control to show the baseline level of Gal-1 in the cells. (B) LLS30 pulls down Gal-1 wild type (WT) but exhibits reduced binding with Gal-1 mutant (D123A) (Mut). Biotin-LLS30 bound to streptavidin-agarose beads was incubated with recombinant proteins Gal-1 WT or Mut. After wash, the level of bound protein was detected via immunoblots with an anti-Gal-1 antibody. Gal-1 WT or Mut alone was loaded as input. (C) The superposition diagrams of the H-Ras(G12V)-Gal-1 complex and the Gal-1-LLS30 (magenta) complexes. (D) The binding region of LLS30 on the surface of the Gal-1 protein is shown in green (left), while the binding region of H-Ras(G12V) on the Gal-1 protein surface is depicted in yellow (middle); the overlapping region where LLS30 and H-Ras(G12V) bind on the Gal-1 protein surface is highlighted in blue (right). (E) Co-immunoprecipitation experiments investigated the interaction between Gal-1 and H-Ras after treatment with LLS30 or vehicle DMSO. F96.2 cells were first treated overnight with either LLS30 or DMSO. Subsequently, cell membrane proteins were extracted, followed by co-immunoprecipitation using an anti-Gal-1 antibody. The input consisted of total membrane protein extracted from NF96.2 cells without co-IP. Both the eluted proteins from co-IP and the total membrane protein without co-IP were analyzed by SDS-PAGE and immunoblotted with an anti-Ras antibody. (F) Immunofluorescence microscopy demonstrating the expression and localization of H-Ras and pERK in NF96.2 cells.

To validate the findings of the docking studies, we performed pull-down assays to verify the physical interaction with Gal-1. Streptavidin-agarose bead bound biotin-LLS30 was prepared and incubated with NF96.2 WCL to pull down interacting proteins. The bound proteins were then eluted and subjected to SDS-PAGE followed by immunoblotting with an antibody against Gal-1. The band that appeared on the immunoblot indicated the presence of Gal-1 in the WCL pulled down by the biotin-LLS30 complex (Fig. 2A), confirming that Gal-1 interacts with LLS30. Furthermore, given that Asp123 plays a significant role in the binding of LLS30 to Gal-1 through hydrogen bond interactions, we introduced a single point mutation (D123A) in Gal-1 to test its importance. The results showed that the D123A mutation reduced binding affinity (Fig. 2B), confirming Asp123’s critical role in the interaction. These findings confirm LLS30’s ability to bind to Gal-1 in MPNST cells, suggesting its potential as a Gal-1 inhibitor for therapeutic application in MPNST. Further binding models analysis showed that both H-Ras(G12V) and LLS30 can interact with residues His52, Ser29 and Asp123 on Gal-1 (Fig. 2C & 2D). This observations suggested that LLS30 has the potential to interfere with the Gal-1/Ras interaction. To test this hypothesis, we used co-immunoprecipitation (co-IP) to examine whether LLS30 reduces the binding of Ras to Gal-1. Indeed, the co-IP results confirmed a reduction in the amount of RAS binding to Gal-1 in the presence of LLS30 (Fig. 2E). Importantly, LLS30 treatment was found to disrupt the association of RAS with the plasma membrane (Fig. 2F). Moreover, phosphorylation of the ERK was found to be significantly reduced after LLS30 treatment (Fig. 2F). Together, these data indicate that LLS30 binds to Gal-1 and disrupts the Gal-1/Ras interaction, leading to the dissociation of Ras from the plasma membrane and a suppression of the Ras/Erk pathway in MPNST cells.

### Anticancer activity of LLS30 against MPNST cells *in vitro*

**Fig 3.**
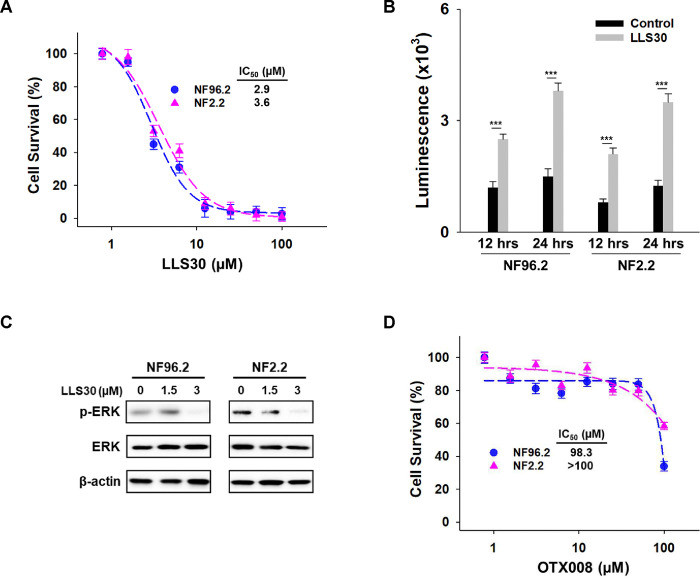
Effects of LLS30 in MPNST cells. (A) Cell survival of NF96.2 and NF2.2 MPNST cells treated with indicated concentrations of LLS30 for 72 hours. IC_50_ of LLS30 on NF96.2 and NF2.2 are 2.9 μM and 3.6 μM, respectively. (B) Caspase-3/7 activities in NF96.2 and NF2.2 cells after 12 and 24 hours of treatment with 0.05% DMSO or 5μM LLS30. (C) Immunoblots of phospho-Erk, Erk and β-actin in NF96.2 and NF2.2 treated with 0, 1.5 or 3 μM LLS30 for 24 hours. (D) Effect of OTX008 on MPNST cell lines. Cell viability of NF96.2 and NF2.2 MPNST cells was assessed after treatment with indicated concentrations of OTX008 for 72 hours. ***p < 0.001; two-tailed Student’s t test. Data shown are mean ± s.d.

Given that LLS30 disrupts Gal-1–Ras interactions, we further evaluated the potential anti-cancer effects of LLS30 on MPNST cells. Proliferation assays showed a significant reduction in MPNST cell growth in a dose-dependent manner after 72 hours of treatment, with IC_50_ values of 2.9 μM against NF96.2 and 3.6 μM against NF2.2 (Fig. 3A). In addition, LLS30 demonstrated the ability to induce apoptosis (Fig. 3B). Given the frequent activation of the Ras/Erk pathway in MPNST patients, we examined whether LLS30 affects this signaling pathway. Treatment with LLS30 at 5μM for 24 hours suppressed the expression of phospho-Erk in NF96.2 and NF2.2 cells (Fig. 3C). Concurrently, we evaluated the efficacy of OTX008, a pre-existing Gal-1 inhibitor tested for treating advanced solid tumors in 2012 (ClinicalTrials.gov: NCT01724320), against MPNST cells. Our data revealed the limited efficacy of OTX008 against MPNST cells (Fig. 3D). Taken together, LLS30 exhibits cytotoxicity against MPNST cells with superior effects compared to OTX008.

### LLS30 has anti-tumor activity in MPNST orthotopic xenograft mouse models

**Fig 4.**
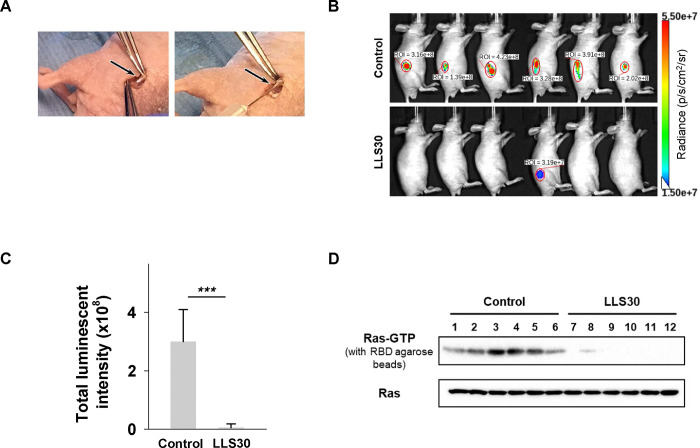
LLS30 suppresses MPNST growth in an orthotopic xenograft mouse model. (A) Illustration of sciatic nerve retrieval for the injection of luciferase-tagged NF96.2 cells to establish the orthotopic xenograft mouse model. (B) Representative bioluminescent imaging at week 8 after LLS30 or vehicle treatment, and (C) quantification of tumor signals. (D) Assessment of activated Ras expression levels using a Ras-GTP pull-down assay and immunoblotted with anti-Ras antibody (upper panel). Total Ras expression in protein extracted from both LLS30-treated and vehicle-treated tumors was examined via immunoblot with anti-Ras antibody (lower panel). Samples in lanes 1–6 were treated with vehicle, while lanes 7–12 received LLS30 treatment. ***p < 0.001; two-tailed Student’s t test. Data shown are mean ± s.d.

We further assessed the impact of LLS30 on MPNST growth *in vivo* by utilizing the orthotopic tumor model, which closely mimics the tumor microenvironment of MPNST. Luciferase-tagged NF96.2 cells were injected into the sciatic nerves of mice (Fig. 4A). Once tumors became palpable in all mice by week 6, they were randomly assigned into two groups and intraperitoneally administered either (1) vehicle or (2) LLS30 (10 mg/kg, once daily for 14 days). Bioluminescence imaging was employed to monitor tumor burden over time. As depicted in Fig. 4B, significant tumor burden was evident in all six control mice by week 8. In contrast, only one mouse in the LLS30-treated group exhibited a limited tumor area (Fig. 4B & 4C). Furthermore, excised tumors were analyzed for activated Ras levels. Active Ras analysis of xenograft tumors revealed that LLS30 treatment reduced the levels of activated Ras (Fig. 4D, upper panel), while total Ras protein in tumor cells remained unaffected (Fig. 4D, lower panel). These studies demonstrate the therapeutic efficacy of LLS30 in inhibiting tumor growth in MPNST orthotopic xenograft mouse models.

### RNA-seq analysis reveals the implication of LLS30 in various cancer pathways

**Fig 5.**
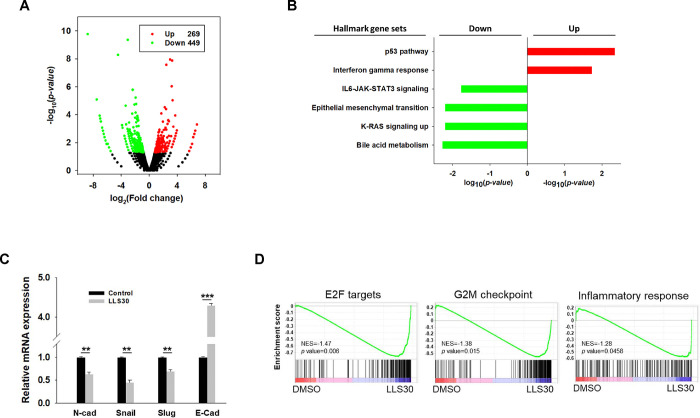
Transcriptomic analysis for LLS30 effects in NF96.2 MPNST cells. (A) Volcano plot. The log_2_ fold change (FC) and log_10_ *p-value* indicated the expression level and significance for each gene. Each dot represents one gene. Black dots represent no significant differential expressed genes between control and LLS30 treatment, the green dots represent downregulated genes (FC<1.5, p<0.05) and red dots represent upregulated genes (FC>1.5, p<0.05). (B) Hallmark gene set analysis of the upregulated genes (FC>1.5, p<0.05) and downregulated genes (FC<1.5, p<0.05) between control and LLS30 treatment. Pathways significantly enriched for upregulated genes are represented by red bars, while those enriched for downregulated genes are denoted by green bars. (C) qRT-PCR analysis of EMT markers, including N-cadherin, Snail, Slug, and E-cadherin, in NF96.2 cells treated with vehicle or LLS30 (5 μM) for 24 hours. (D) Gene set enrichment analysis (GSEA) of hallmark gene sets significantly enriched in LLS30 treated cells vs control, with NES indicating nominal enrichment score. A gene set shows significant enrichment at a *p* < 0.05. **p < 0.01, ***p < 0.001; two-tailed Student’s t test. Data shown are mean ± s.d.

In addition to establishing that LLS30 interferes with the Gal-1/RAS interaction, we conducted a comprehensive investigation into the gene regulatory processes involved in LLS30-induced cell death. We examined RNA-Seq datasets to identify functional enrichments among differentially expressed genes in NF96.2 cells. RNA-Seq analysis revealed that LLS30 treatment resulted in the upregulation of 269 genes and the downregulation of 449 genes, each with at least a 1.5-fold change and *p* < 0.05 (Fig. 5A). Functional pathway analysis of these differentially expressed genes indicated that LLS30 affects multiple important cellular pathways. In the Hallmark database, the upregulated genes were involved only in the P53 (*p* = 4.5E-03) and interferon gamma (IFNγ) (*p* = 1.8E-02) pathways (Fig. 5B). In addition, the downregulated pathways included bile acid metabolism, KRAS signaling, epithelial-mesenchymal transition (EMT), and IL6-JAK-STAT3 signaling (Fig. 5B). qRT-PCR validated the suppression of EMT markers by LLS30, showing decreased levels of N-cadherin, snail, and slug, and increased levels of E-cadherin (Fig. 5C). Furthermore, GSEA using the Hallmark gene set collection revealed that LLS30 treatment significantly disrupted proliferation-related pathways including E2F targets (*p* = 6E-03) and the G2M checkpoint (*p* = 1.5E-02), as well as an inflammatory response (*p* = 4.6E-02) compared to the control group treated with DMSO (Fig. 5D). These findings suggested that LLS30 not only suppresses cancer progression by regulating multiple signaling pathways but also potentially inhibits metastasis through the regulation of EMT.

### LLS30 inhibits formation and growth of experimental MPNST metastasis

**Fig 6.**
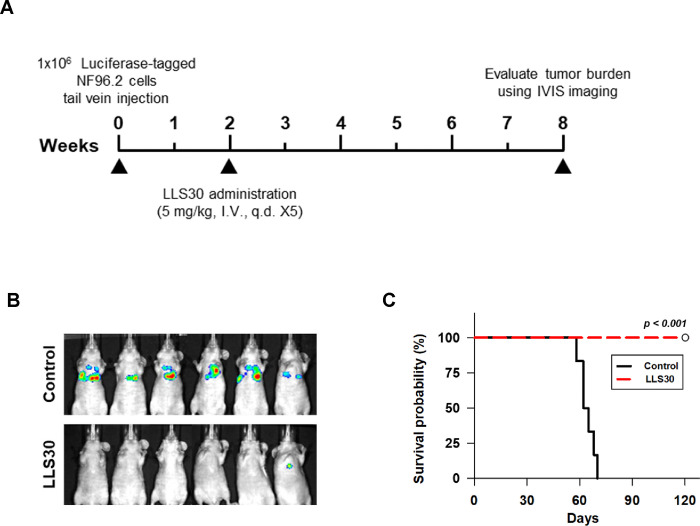
NF96.2 lung metastases bearing mice were treated with LLS30. (A) The timeline illustrates the LLS30 treatment protocol. (B) Bioluminescent images were captured for control (8.7% alcohol/8.7% Tween-80) or LLS30-treated mice at 8 weeks after the initial i.v. injection of luciferase-tagged NF96.2 cells. (C) The Kaplan-Meier plot depicts the survival of PBS (n = 6) and LLS30-treated mice (n = 6). ***p < 0.001; log-rank test.

MPNSTs, aggressive and highly metastatic sarcomas arising from the myelinating nerve sheath, often exhibit metastasis, primarily to the lung, within 2 years of initial disease presentation(26). Noticeably, our RNA-seq analysis shows that LLS30 treatment downregulated the EMT signaling (Fig. 5B, 5C), which is well-known for its association with tumor invasion and metastasis in cancer(27). This observation prompted us to assess whether LLS30 inhibits MPNST cell colonies formation in the lung using our established experimental metastasis model(23). Luciferase-tagged NF96.2 cells were injected into the tail vein, and lung colonies were observed two weeks after tumor cell injection. At this point, LLS30 was administered at a lower dose of 5 mg/kg to mitigate its effect on cell proliferation, with the treatment duration spanning five days. By week 8, macroscopic lung metastases were evident in all 6 control mice (Fig. 6B). Conversely, among the LLS30-treated group, no visible metastases were detected in five mice, while one exhibited a slight signal (Fig. 6B). Importantly, LLS30 treatment significantly improved survival (*p* < 0.001 compared to control; Fig. 6C). While all control mice had died by day 70, all LLS30-treated mice remained alive at 120 days. These results demonstrate that LLS30 treatment inhibits the growth of MPNST lung metastases, leading to a significant increase in survival.

## Discussion

The pivotal role of RAS in malignancies makes them as the primary targets for cancer therapies. Despite extensive efforts, attempts to target RAS and develop clinically approved drugs have proven unsuccessful. Given that RAS proteins require membrane association for their biological activity, disrupting this association emerges as a viable strategy for cancer treatment. In this study, we confirmed the role of LLS30 as a Gal-1 inhibitor for treating MPNST, demonstrating that LLS30 disrupts the Gal-1/Ras interaction, leading to Ras dissociation from the plasma membrane and subsequent suppression of Ras activation. Our study provided valuable insights into the molecular mechanisms underlying the anti-cancer effects of LLS30 and highlighted its promise as a therapeutic agent for MPNST treatment.

Significant research has explored Gal-1 inhibition in experimental cancer models. Thiodigalactoside has shown efficacy in slowing breast cancer progression when combined with vaccine immunotherapy(28–31). OTX008, a stable small molecule Gal-1 inhibitor, disrupts ERK signaling and induces G2/M cell cycle arrest in various human cancer cell lines(32). Preclinical data suggested OTX008’s potential efficacy against solid tumors alone or in combination with standard treatments(33, 34). Despite a 2012 clinical trial evaluating OTX008 for advanced solid tumors, its outcome remains undisclosed. This underscores the limited effectiveness of existing Gal-1 inhibitors for human use and the urgent need for safer and more effective alternatives. In this study, preclinical evidence showed that LLS30 inhibited tumor growth and metastasis in MPNST mouse models. To our knowledge, our team is currently the sole entity examining Gal-1 inhibition through LLS30 specifically in the context of MPNST. Our findings expand the scope of Gal-1 inhibitors, paving the way for novel therapeutic strategies in the management of MPNST.

Increased evidence suggests that tumor-intrinsic signaling can influence the immune response to the tumor(35). Generally, tumors with the presence of tumor infiltrated lymphocytes (TILs), expressions of PD-L1 and tumor-related immune cells, possible genomic instability, and the presence of a pre-existing antitumoral immune response (“hot” tumors) are typically respond well to immunotherapy. In contrast, “cold” tumors, which lack these features, generally require treatments that transform them into “hot” tumors to improve their response to immunotherapy. MPNST is considered immunologically ‘cold’ tumors with minimal T cell infiltrates(36, 37). In this study, following LLS30 treatment, we observed the upregulation of IFNγ, a potent switch and enhancer for the recruitment of innate immune cells. This exciting result suggested that LLS30 may have the potential to transform immunologically “cold” tumors into “hot” ones by upregulating IFNγ. Consequently, this could increase TILs and enhance the MPNST response to PD-1 checkpoint inhibitor therapies. However, there have been no published *in vivo* studies exploring immunotherapy regimens tailored specifically to MPNST. We’re currently actively investigating immunotherapy for MPNST, specifically focusing on examining the combined effects of LLS30 and PD-1 checkpoint inhibitors for treatment.

There has been increasing recognition of the role of EMT in cancer pathogenesis, including tumor initiation, invasion, metastasis, and therapy resistance(38). In MPNST, Wang et al. demonstrated that low expression of protein tyrosine phosphatase receptor S (PTPRS) correlates with poor prognosis, suggesting its function as a tumor suppressor(39). Knockdown of PTPRS induces EMT, characterized by increased levels of N-cadherin and alpha smooth muscle actin (αSMA), and decreased levels of E-cadherin. In addition, upregulation of profilin 1, an actin-binding protein (ABP), is observed in PTPRS-downregulated MPNST cells, promoting EMT-mediated motility and invasion. Restoration of EMT processes induced by PTPRS downregulation is achieved through the downregulation of profilin 1. Consistent with this finding, our research demonstrates that LLS30 treatment downregulates profilin 1. Furthermore, using pulmonary metastasis models of MPNST, we showed that LLS30 treatment significantly reduces lung metastasis *in vivo*.

In conclusion, our comprehensive analysis demonstrates that LLS30 treatment not only interferes with Gal-1/RAS interaction but also induces significant alterations in gene regulatory processes, including modulation of key cellular pathways such as P53, interferon gamma, and proliferation-related pathways, while disrupting EMT. Importantly, LLS30 significantly inhibited tumor growth and metastasis in MPNST mouse models. These findings emphasize the importance of further research and development of LLS30 for potential translation into human clinical trials, offering hope for improved management of MPNST.

## Figures and Tables

**Table 1.** The contact list between Gal-1 with H-Ras(G12V).

Chain 1	Residue	Chain 2	Residue	Interaction type

H-Ras(G12V)	Gln25.CA	Gal-1	Cys2.SG	Hydrogen bond interaction
H-Ras(G12V)	Tyr32.OH	Gal-1	Asp123.OD1	Hydrogen bond interaction
H-Ras(G12V)	Thr35.OG1	Gal-1	Asn33.OD1	Hydrogen bond interaction
H-Ras(G12V)	Glu37.OE1	Gal-1	His52.ND1	Salt bridge
H-Ras(G12V)	Asp38.OD1	Gal-1	Ser29.OG	Hydrogen bond interaction
H-Ras(G12V)	Asp38.OD1	Gal-1	His52.NE2	Salt bridge
H-Ras(G12V)	Tyr40.OH	Gal-1	Asp123.OD1	Hydrogen bond interaction
H-Ras(G12V)	Arg41.NH2	Gal-1	Asp26.OD2	Salt bridge
H-Ras(G12V)	Glu63.OE1/OE2	Gal-1	Lys63.NZ	Salt bridge
H-Ras(G12V)	Glu63.OE1	Gal-1	Ala67.CA	Hydrogen bond interaction
H-Ras(G12V)	Glu63.OE2	Gal-1	Trp68.NE1	Hydrogen bond interaction
H-Ras(G12V)	Met67.N	Gal-1	His52.O	Hydrogen bond interaction

## Data Availability

The datasets generated and/or analysed during the current study are available from the corresponding author on reasonable request.
